# Age-Dependent Statistical Changes of Involuntary Head Motion Signatures Across Autism and Controls of the ABIDE Repository

**DOI:** 10.3389/fnint.2020.00023

**Published:** 2020-06-17

**Authors:** Carla Caballero, Sejal Mistry, Elizabeth B. Torres

**Affiliations:** ^1^Sports Research Center, Sports Sciences Department, Miguel Hernández University of Elche, Elche, Spain; ^2^Department of Psychology, Rutgers, The State University of New Jersey, Piscataway, NJ, United States; ^3^Department of Mathematics, Rutgers, The State University of New Jersey, Piscataway, NJ, United States; ^4^Computer Science, Center for Computational Biomedicine Imaging and Modeling, Rutgers, The State University of New Jersey, Piscataway, NJ, United States; ^5^Center for Cognitive Science, Rutgers, The State University of New Jersey, Piscataway, NJ, United States

**Keywords:** autism spectrum disorder, involuntary motions, stochastic analyses, head motion analysis, resting state – fMRI, Gamma distributed data

## Abstract

The DSM-5 definition of autism spectrum disorders includes sensory issues and part of the sensory information that the brain continuously receives comes from kinesthetic reafference, in the form of self-generated motions, including those that the nervous systems produce at rest. Some of the movements that we self-generate are deliberate, while some occur spontaneously, consequentially following those that we can control. Yet, some motions occur involuntarily, largely beneath our awareness. We do not know much about involuntary motions across development, but these motions typically manifest during resting state in fMRI studies. Here we ask in a large data set from the Autism Brain Imaging Exchange repository, whether the stochastic signatures of variability in the involuntary motions of the head typically shift with age. We further ask if those motions registered from individuals with autism show a significant departure from the normative data as we examine different age groups selected at random from cross-sections of the population. We find significant shifts in statistical features of typical levels of involuntary head motions for different age groups. Further, we find that in autism these changes also manifest in non-uniform ways, and that they significantly differ from their age-matched groups. The results suggest that the levels of random involuntary motor noise are elevated in autism across age groups. This calls for the use of different age-appropriate statistical models in research that involves dynamically changing signals self-generated by the nervous systems.

## Introduction

The volitional control of physical movements, i.e., the control of our purposeful actions at will, and the healthy preservation of this ability, are fundamental elements to generate well-coordinated behaviors across the human lifespan. As the somatic-sensory-motor systems of human babies mature and give way to several developmental milestones, spanning from infancy to the elderly stages of our life cycle, the patterns of variability in our motions are bound to change (Torres et al., 2016b). These changes reflect the outputs of our nervous systems and can be a valuable tool to track healthy neurodevelopment and healthy aging in contrast to neurodevelopmental differences and neurodegeneration.

One of the signs of motor dysfunction that appears later in life is the abundance of undesirable involuntary motions. When a person is asked to remain still, there is (inevitably) some level of involuntary micro-motions across the body; yet if such levels are persistently high in early neurodevelopment, they can interfere with neuromotor control and forecast upcoming problems with the nervous systems. They can predict problems with action coordination and volitional control of goal directed behaviors (Torres et al., 2013a; Wu et al., 2018), but these are difficult to detect using traditional statistical analyses based on grand averaging under assumed Gaussian distribution [as explained in Torres et al. (2013a) situating autism within the broader context of Precision Medicine].

The study of evolving trends in the self-generation of undesirable involuntary motions at the periphery (Brincker and Torres, 2013), along with their variable rates of change across the human lifespan, requires age-appropriate adjustments of our statistical analyses across different aging human populations. This include for example, size-dependent (allometric) standardizations of data harnessed from different anatomies owing to different ages (Mosimann, 1970; Lleonart et al., 2000). There is, however, a paucity of studies reflecting the cross-sectional age-dependent evolution of the variability in motor patterns contributing to volitional control for neurotypicals. In the absence of such normative data to characterize patterns of motor variability in healthy early neurodevelopment and in the aging population, most statistical analyses of human behaviors are performed under a *one-size-fits-all* approach that uses parametric statistics and linear models. This treatment of the problem may prevent us from considering the non-linear complex dynamics of biorhythmic activities produced by the developing and the aging nervous systems.

While other fields have considered various non-linear models, e.g., of heart rate variability (Peng et al., 1995) and gait patterns (Raffalt et al., 2018; Caballero et al., 2019), the focus of that work has been on suitable methods to assess both long-range and short-range correlations in non-stationary and stationary systems. The data that interests us here is brief, limited by the number of frames in a scanning fMRI session, during resting state, when the person has been asked to remain still. As such, our interest focuses on the nature of the families of distributions that we could empirically derive from fluctuations of involuntary bodily motions across different age groups of the neurotypical and autistic populations. More specifically, we assess the extent to which such families of distributions may typically shift cross sectionally in the neurotypical population. Our approach contrasts with traditional approaches that make *a priori* theoretical assumptions on the nature of such distributions and tend to obfuscate our abilities to predict possible departures from normative states in pathological states of the nervous systems, where asynchronous attainment of developmental milestones abound. One such example is evidenced in research involving autism spectrum disorders (ASD).

**FIGURE 1:**
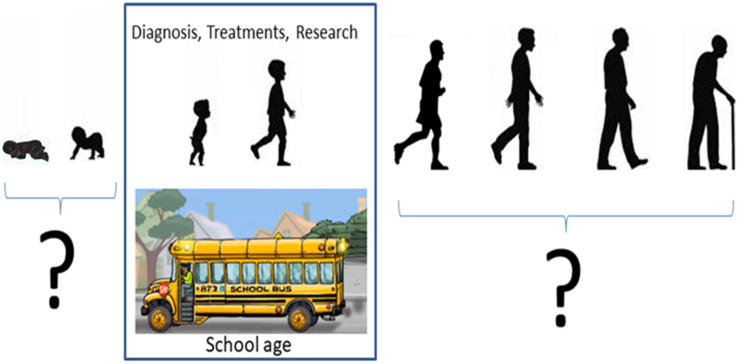
Science has very limited knowledge of autism as a lifelong condition. Research in autism has been focused on certain age groups primarily involving children of school age. We know very little about neurodevelopment preceding autism and virtually nothing about adults. Parents ask, “*what will happen to my child when the yellow bus stops coming*,” as in the US, services taper off as children transition into adulthood (a phase that parents have coined “*falling off the cliff*”). No proper methods have been designed to study neuromotor issues of aging adults with autism, often presenting ataxia syndromes, loss of balance, frequent falls and symptoms of Parkinsonism (Starkstein et al., 2015).

Autism is a lifelong, highly heterogeneous, evolving condition (Lord et al., 2000; Constantino and Charman, 2016) and yet, we know very little about maturational patterns of somatic-sensory-motor signatures, critical to scaffold the volitional control of the brain over the body in motion. Understanding such differences in the peripheral input to central motor control across the population is important in more than one way. From the research standpoint, such peripheral patterns have been revealing of maturational stages and possible familial ties (Torres et al., 2013a, 2016a; Wu et al., 2018) amenable to help us further our understanding of the etiology of the condition, trace back the individual contextual and environmental features of the developmental trajectories of each person, and tailor treatments and services according to family needs, in a personalized manner. From the societal standpoint, it is important to know the ever-changing needs of the person’s level of motor autonomy, to advocate for public policies that help to effectively deploy and manage resources that support the development of independent living prior to and beyond school age (Figure 1). Given the heterogeneity of ASD statistics (Torres et al., 2016a), there is a critical need to stratify the affected population and design interventions that are age-appropriate, personalized to the person’s needs and congruent with the profound differences that define the somatic-sensory-motor profiles characterizing the autistic phenotype, e.g., (Lancaster et al., 2013; Torres et al., 2013a; Marko et al., 2015; Mosconi and Sweeney, 2015; Mosconi et al., 2015a; Sharer et al., 2015; Mahajan et al., 2016; Torres and Denisova, 2016; Chuye et al., 2018).

Designing new ways to uncover self-emergent clusters of data to stratify the heterogeneous ASD population has been rather challenging, owing this in part to the lack of access by most researchers to participants of diverse ages, and to the lack of data that is inclusive of both sexes. The advent of open access ASD and typically developing (TD) data repositories addresses these issues today and enables us to explore the question of age-dependent shifts in the signatures of variability across the normative data from the neurotypical population and the population with an ASD diagnosis. One such open access databases is the Autism Brain Imaging Data Exchange (ABIDE) repository (Di Martino et al., 2014) an effort that has revealed several new features of brain organization (Alaerts et al., 2014; Ray et al., 2014; Plitt et al., 2015; Haar et al., 2016), new differentiating features of females (Torres et al., 2017) and males (Supekar and Menon, 2015; Jung et al., 2019), IQ and medication intake (Torres and Denisova, 2016) and patterns of scanner-dependent noise in the involuntary head motions (Caballero et al., 2018). This has been part of a general effort to understand neuromotor control in ASD (Ornitz, 1974; Minshew et al., 2004; Mandelbaum et al., 2006; Perry et al., 2007; Jasmin et al., 2009; Kushki et al., 2011; Donnellan et al., 2012; Torres et al., 2013a; Hannant et al., 2016). In ABIDE, it is possible to use the imaging data and extract head motions in the form of rotations and displacements (a routine step in removing motor artifacts from the images) such that the extracted involuntary head micro-motions when the person is trying to remain still, can give us a sense of the amount of volitional control that people typically have across different age groups. In turn, given that ABIDE has age- and sex- matched participants with ASD, we can interrogate the database across different age groups, to learn about age-dependent shifts in the statistics of undesirable involuntary head motions.

In this paper, we explore data in ABIDE, to characterize statistical patterns of involuntary head motions across ages, as the person is instructed to remain still and yet the data reveal undesirable involuntary head motions. We compile the imaging data to extract the patterns of head translation and rotation across each session and use these time series (waveforms) of the linear and angular speed to characterize differences in volitional control as an inevitable feature, preventing the person from remaining still at will. We ask if the stochastic signatures derived from the patterns of head motion variability differ across ages in the neurotypical population. We further ask if the participants with ASD depart from the normative signatures.

## Materials and Methods

### Demographics of ABIDE I and II

All datasets included in this study are from the Autism Brain Imaging Data Exchange (ABIDE) databases: ABIDE I^1^ and ABIDE II^2^. ABIDE obeys the following guideline on the use of human subject’s data: “In accordance with HIPAA guidelines and 1000 Functional Connectomes Project/INDI protocols, all datasets have been anonymized, with no protected health information included.”

The study includes two main comparisons:

(1) *Autism Spectrum Disorder (ASD)*, and *Typical Development (TD)*, using estimation of stochastic signatures of involuntary head micro-movements of individuals with a formal DSM-ASD (American Psychiatric Association, 2013) diagnosis of ASD and TD controls.
(2) *Ranges of age*. Each group (ASD and TD) was split in seven different groups according to their age to assess how the stochastic signature of involuntary head micro-movements evolves with growth. The ranges of age used to that end were the following: from 5 to 10 years old, from 11 to 15 years old, from 16 to 20 years old, from 21 to 25 years old, from 26 to 30 years old, from 31 to 40 years old, and from 41 to 65 years old.

### Inclusion/Exclusion Criteria

This study includes all sites publicly available through ABIDE I and ABIDE II. They were comprised of 1,127 TD and 1,017 ASD. As we explained above, those groups were divided by age. Table 1 provides the number of participants with ASD or TD are in each range of age in ABIDE dataset.

**TABLE 1:** *t*-test *p*-values comparing the cumulative linear and angular excursions for ASD vs. TD in each age group (yo stands for years old).

**Age group**	**5–10 yo**	**11–15 yo**	**16–20 yo**	**21–25 yo**	**26–30 yo**	**31–40 yo**	**41–60 yo**
Cum Lin Speed	1.00 10^–12^	1.00 10^–12^	1.00 10^–12^	1.00 10^–12^	1.00 10^–12^	0.70 10^–12^	1.00 10^–12^
Cum Ang Speed	1.00 10^–4^	1.00 10^–4^	1.00 10^–4^	1.00 10^–4^	1.00 10^–4^	0.44 10^–4^	1.00 10^–4^

### Bootstrapping Method

The analyses referring to the bootstrapping methods were previously published but we will refer to them here for simplicity.

First, we uniformly resampled all data sets to avoid temporal inconsistencies, since our focus is on fluctuations in signal amplitude. To that end, we resample all data to ensure equally spaced points for comparison across subjects and groups (outcome can be seen in Supplementary Material of prior work^3^). We use the MATLAB (version R2014a, The MathWorks, Inc., Natick, MA, United States) function resample which applies an antialiasing FIR low-pass filter to the time series and compensates for the delay introduced by the filter. This function resamples the input sequence, the raw head motion in our case, at P/Q times the original sample rate [see Supplementary Table S1 of the previously published SM for more information about the resampling factors used (P and Q)].

Second, we apply uniform data length by truncating the uniformly resampled data to ensure the same length for all the time series.

Given the inconsistent group sizes extracted from the ABIDE datasets (see Table 1), we used a bootstrapping method previously described to ensure uniform group numbers for pairwise statistical comparisons across ages. To that end, we used random sampling with replacement and created 100 subgroups drawn from the original size group while considering the minimum number *n* = 25 at a time. These 25 randomly selected participants’ data contribute to a data point in the age group of 100 participants. Their head motion time series are pooled to further create a standardized waveform, free of allometric effects from different anatomical sizes and focusing on the variability patterns relative to the overall empirically estimated mean speed amplitude expressed by the group. We chose 25 as the size to randomize because the smallest age’s sub-groups size was *n* = 30. Thus, after dividing the groups by age, we extracted the 100 random sub-groups with replacement, using the same size (*n* = 25) to make up 100 group sizes from all the age’s sub-groups. Supplementary Material Figures A4, A5 show the results from sampling without replacement.

### Data Processing

#### Motion Extraction

Head motion patterns were extracted from imaging data during (rs) fMRI experiments. Motion extraction was performed using the Analysis of Functional NeuroImages (AFNI) software packages (Cox, 1996). Single-subject processing scripts were generated using the afni_proc.py interface^4^. Skull stripping was performed on anatomical data and functional EPI data were co-registered to anatomical images. The median was used as the EPI base in alignment. Motion parameters, 3 translational (*x*-, *y*-, and *z*-) and 3 rotational (pitch-about the *x*-axis, roll-about the *y*-axis, and yaw- about the *z*-axis), from EPI time-series registration was saved.

We note the caveat that different labs depositing data in ABIDE may use different padding to restrain/dampen head motion in general. However, each site of ABIDE has deposited data from a similar scanner and padding method for controls and autistics. We used the bootstrapping method to shuffle the fluctuations in speed amplitude and emphasize here that these fluctuations in speed amplitude that we examine are relative to an empirically estimated mean head motion speed (linear mm/s or angular rad/s). These data do not refer to the absolute value of the speed which may be differentially affected by the type of padding.

### Head Excursion

To obtain the head excursions we accumulate the distance traveled per unit time (speed) and determine the pathlength of the linear displacement. We also determine the full excursion yielded by the accumulation of angular displacements. These parameters give us a sense of the net amount of physical head motion a person had while instructed to try to remain still. In both cases, we used the same number of data points for each participant, yet across those frames, each participant varied in the rate of change of displacements and their accumulation over time.

### Statistical Analyses

We describe two components of the analyses of the head motions: (1) The standardized data type called micro-movement spikes, MMS and (2) the statistical platform for individualized behavioral analyses (SPIBA), both previously defined (Torres et al., 2013a) and US Patented methods publicly available^5^.

In the present work, we assess the scan-by-scan speed-dependent variations in the amplitude of the linear displacement (mm/s) and in the angular rotations (rad/s) of the head relative to the empirically estimated mean of each person (personalized method) during resting-state functional magnetic resonance imaging (rs-fMRI) sessions. The analyses specifically refer to the stochastic signatures of MMS [defined in prior peer reviewed work including earlier versions of the ABIDE data and of others data sets (Torres et al., 2016a, 2017; Torres and Denisova, 2016; Caballero et al., 2018)].

### Micro-Movement Spikes

**FIGURE 2:**
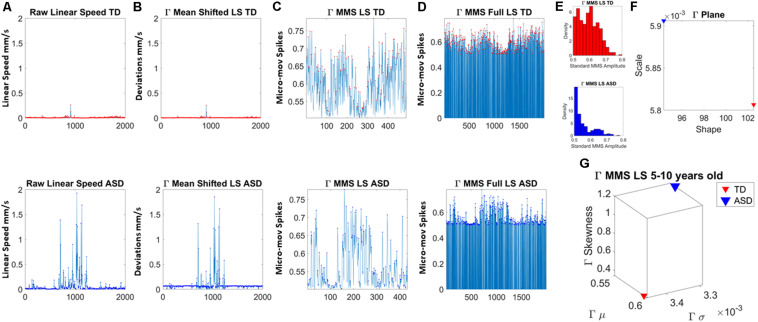
Analytical pipeline. **(A)** Sample time series of involuntary head motion expressed as the linear speed (mm/s) derived from linear head displacements from a representative typically developing (TD) participant (top) and a representative participant with ASD (bottom) with equal number of frames for all participants. **(B)** Absolute amplitude deviations from the empirically estimated Gamma mean (empirically estimated shape × scale) amplitude. **(C)** Gamma micro-movement spikes, MMS, obtained from the deviations from the mean by normalizing the waveform to account for allometric effects. Each peak is divided by the sum of the peak value and the average value of the values comprised within the local minima adjacent to the peak (inclusive of the local minima). **(D)** All MMS embedded in the original waveform across all frames. **(E)** The MMS peaks are gathered in a frequency histogram. **(F)** The maximum likelihood estimation method is used to determine the continuous family of distributions best fitting both data sets; then the empirically estimated shape and scale values are plotted on the Gamma parameter plane. **(G)** The corresponding Gamma moments are plotted on a parameter space that includes the mean, standard deviation, skewness and kurtosis to aid visualize the signature of each participant and localize TD and ASD on these parameter spaces.

The maximum amplitude of the speed (linear *mm/s* and angular *deg/s*) was obtained from the raw data extracted from the head motions (Figure 2A). The empirically estimated mean speed of each person was also obtained and used as reference to determine the maximal amplitude deviations from it (Figure 2B). The time-series of these fluctuations in maximal amplitude deviations from the empirically estimated mean provides the waveform of interest for our analyses. These are the spike trains of random fluctuations in signal amplitude (speed in this case). The fluctuations in amplitude of those spikes are normalized between [0,1] and used as continuous spike trains with amplitude values in the real domain. More generally, they are treated as an identically independent distributed (iid) continuous random process using the time series forecasting analytical framework (Hamilton, 1994), where events in the past may (or may not) accumulate evidence toward prediction of future events.

In this work, to remove allometric effects of body-size across ages in each trial we computed the normalized peak amplitude (the peak speed amplitude is divided by the sum of the peak speed amplitude and the averaged speed amplitude value comprising points between the two speed minima surrounding the local peak amplitude) (Mosimann, 1970; Lleonart et al., 2000). The normalized fluctuations define the micro-movement spikes of the original speed waveform. These are shown in Figure 2C. Figure 2D shows the MMS as they occurred in the original waveform, thus preserving the original number of frames. This waveform is amenable to perform other analyses (e.g., pairwise cross-coherence, pairwise cross-correlation, etc. to understand the periodic behavior of the MMS of a given biorhythm).

In the specific case of rs-fMRI data here, the data types used in this work are not the original head motions *per se*, but rather derivative information pulled out from the original time series that the head-motion extraction methods create. The commonly used methods to estimate volume-to-volume head movement from fMRI data were used here to obtain the original time series of (raw) head motion data (see section on “Materials and Methods” for head motion extraction above). Importantly ABIDE has two versions of the data sets, one which has been cleaned from artifacts and one which is raw (uncleaned). Since we are precisely interested in the continuous acquisition of head motion, we used the uncleaned data sets. Note also that in an effort to reproduce our results, every publication does report to ABIDE the indexes of the data that has been used in the analyses. As such, we report to ABIDE the indexes used in this work.

To ascertain the net physical head motions across all participants, we compute the cumulative distance traveled per unit time and this gives us the path length of the linear and angular displacements (as explained above). The empirically estimated mean was obtained using the continuous Gamma family of probability distributions for every group [as in Torres and Denisova (2016), Torres et al. (2017), Caballero et al. (2018) because it gave the best fit according to maximum likelihood estimation, MLE] (see Table 2 for information about the mean head excursion for every group).

**TABLE 2:** Glass Delta and Cohen *d* values to quantify disease effect.

**Age group**	**5–10 yo**	**11–15 yo**	**16–20 yo**	**21–25 yo**	**26–30 yo**	**31–40 yo**	**41–60 yo**
**Head’s linear excursion (displacements)**							
Glass Delta	2.76	3.24	3.17	2.34	5.44	1.32	1.17
Cohen *d*	1.30	1.80	2.28	1.68	1.71	0.59	3.53
**Head’s angular excursion (rotations)**							
Glass Delta	2.09	2.94	3.38	2.12	2.12	0.55	4.09
Cohen *d*	1.30	1.81	2.28	1.69	1.71	0.59	3.53

In our prior work, the MMS generally served as input to a Gamma process under the general rubric of Poisson random process. We more specifically adapted methods from cortical spike analyses commonly used in the field of computational neuroscience, to analyze fluctuations in biorhythmic data from natural behaviors. Such data are lengthy time series of different physical units registered using different instruments. A such, they are disparate in frequency and timing, and no unifying platform existed to enable the analyses of multiple levels of neuromotor control co-registered with different instruments. We created a unitless data type amenable to combine data from different modalities (e.g., EEG in microVolts, ECG inter beat intervals in ms, EMG in volts, kinematics in m, m/s, m/s^2^, rad, rad/s, rad/s^2^, etc.) and paired this data type with methods to derive other parameterizations of the nervous systems output under different control regimes (voluntary, involuntary, and autonomic). These regimes are grounded on our proposed phylogenetically orderly taxonomy of neurodevelopmental maturation involving three fundamental muscle types (*skeletal muscle, smooth muscle, and cardiac muscle*) associated with specific genes and proteins that would eventually enable us to stratify heterogeneous disorders of the nervous systems using a combination of objective (digitally obtained) behavioral and genetic information. Among these disorders are Parkinson’s disease, the Ataxias, Traumatic Brain Injury and Autism Spectrum Disorders, the latter being of interest in the present work.

In this paper, we specifically focus on involuntary head motions to assess the distribution fitting of the frequency histograms of the time series of their peaks for each age group. We used the stochastic characterization of fluctuations in peaks’ amplitude to characterize the signature of involuntary head motions in the ASD vs. TD groups cross sectionally, across different ages. The motivation here is to estimate the spike trains’ randomness and their levels of noise to signal ratio using the family of distributions best fitting the frequency histograms of the peaks accumulated from the MMS of each individual member of an age group.

We used maximum likelihood estimation, MLE to approximate the best fitting distribution encompassing all cases. To that end, we compared different families of probability distributions (e.g., the Gaussian, Lognormal, Exponential, and Gamma, although the MLE selection criterion does not penalize models with a larger number of parameters -in our case Exponential having one parameter, and other distributions two).

The motivation for these distributions came from prior work in our lab discovering the presence of the Exponential distribution in biorhythms of the autistic *peripheral nervous systems* (Torres, 2011a,b). Controls up to then had been well characterized by the Lognormal family using a multiplicative random process (Ross, 1996), as heavy tailed distributions were near symmetric after log transforming the original speed data. The presence of the Exponential distribution in autistic peripheral signals prompted us to use instead an additive random process. We tried the continuous Gamma family of distributions, which includes the Exponential case when the shape parameter is 1 (as it was in Autism for linear speed peaks.) Another distribution was the Gaussian, to compare the outcome of MLE with the traditional assumption. In all cases, we estimated as well the 95% confidence intervals for the shape and for the scale parameters. The Supplementary Material from our prior work with ABIDE data showed the use of MLE and our finding that the continuous family of Gamma distributions was the best fit. The reader can find these explanations in detail within the Supplementary Material in those papers using these ABIDE sets^6^.

The estimated parameters were plotted on a Gamma parameter plane, where the *x*-axis represents the shape parameter value and the *y*-axis represents the scale parameter value. Figure 2E shows the frequency histogram of sample data from two representative participants, while Figure 2F shows the sample empirically estimated Gamma parameters plotted on the Gamma parameter plane.

The Gamma scale value conveys the *noise to signal ratio* (NSR) since the Gamma mean μ_Γ_ = *a*⋅*b* and the Gamma variance is σ_Γ_ = *a*⋅*b*^2^, thus the scale is:

$$b=\frac{\sigma_{\Gamma }}{\mu_{\Gamma }}=\frac{/a\cdot b^{/2}}{/a\cdot /b}$$

In this sense, the Gamma parameter plane allows us to infer speed-dependent processes leading to higher noise levels vs. lower noise levels. Further, since higher shape values tend toward symmetric distributions and lower values tend to be skewed distributions, with the extreme Exponential distributions at *a=1*, we can also track processes that tend to the Exponential (memoryless, most random) vs. processes that tend toward the Gaussian distribution (more predictable at low NSR).

The scatter of points on the log–log Gamma plane uncovers a power-law relation between the shape and the dispersion of the distributions [the scale parameter or Noise-to-Signal Ratio (NSR)]. The Supplementary Material Figure A7 (TD) and Supplementary Material Figure A8 (ASD) show this and tabulates the fitting errors of the linear polynomial fit with the slope and intercept estimated for each age group and for the pooled data, with 95% approximated confidence intervals. We note that this linear fit is only the case upon the normalization presented here to account for allometric effects owing to different anatomical sizes across different ages. If the raw speed peaks are used instead, this power law relation does not hold. Further, other normalizations (e.g., scaling by dividing by the maximum amplitude) do not hold a power law either. In our experience the ASD data has systematically higher fitting error than the TD data.

In addition, for visualization purposes and to quantify differences in probability space, we compute the empirically estimated Gamma moments (mean along the *x*-axis, standard deviation along the *y*-axis, skewness along the *z*-axis and kurtosis proportional to the size of the marker). These are then plot, for each participant in each age group. Figure 2G shows an example for the representative TD vs. ASD participants used here to illustrate the analyses pipeline. We also plot the Gamma Probability Density Functions (PDFs) using the empirically estimated parameters.

### Statistical Comparison

We used the Kruskal–Wallis non-parametric ANOVA to compare groups pairwise and report in each pairwise comparison the results for *p* < 0.01 and *p* < 0.05 in matrix form, without correction for multiple comparisons. A 7 × 7 matrix of 7 age groups provides the entries with *p*-values (see color bar in figures) and indicates the level of significance: one asterisk for *p* < 0.05 and two asterisks for *p* < 0.01. There are three such matrices, one for comparisons within the group of neurotypicals, one within the group of autistics and one comparing autistic relative to neurotypicals.

The distributions PDFs were also compared using the Kolmogorov–Smirnov test for two empirically estimated distributions and significance reported as above in matrix form. As with the non-parametric ANOVA we report *p*-values as entries of the matrix with one asterisk reflecting significance at 0.05, while two asterisks reflect significance at the 0.01 level.

### Effect Size

In addition to the non-parametric one-way ANOVA (Kruskal–Wallis test), to assess the statistical significance of the group differences, we performed a *t*-test and ascertained the effect size of the differences that these comparisons yielded. To that end, we used the Cohen d test. We also used the Glass delta test, as the samples had equal size but significant differences in their variances. We used the head excursions [the cumulative linear (and the angular) speed] as the parameter of interest and set the neurotypical participants as the control group. The motivation for this parameter is that it is the parameter underlying the MMS computation, as they are derived from the head linear speed and the head angular speed, and we are interested in the cumulative effects over time, along these time series data.

The Cohen *d* test has the following formula:

_d=(M_2_-M_1_)/*SD*_*pooled*__ where *M*_1_ and *M*_2_ are the means of each group,

SD⁢and1⁢SD⁢are2⁢the⁢standard⁢deviations⁢of⁢each⁢group,

$$\mathrm{and}\,{\mathrm{SD}}_{\mathrm{pooled}}=\sqrt{\left({\mathrm{SD}}_{1}^{2}+{\mathrm{SD}}_{2}^{2}\right)/2}.$$

The Glass delta test is Δ = (*M*_1_−*M*_2_)/*SD*_2_ where SD_2_ is the standard deviation of the control group.

We obtained these measurements for each of the 7 age-groups and within each case, compared ASD vs. TD, with TD set as the control group.

The literature (Cohen, 1992; Sawilowsky, 2003) suggests the following size effect ranges: 0.01 very small; 0.2 small; 0.5 medium; 0.8 large; 1.2 very large; and 2.0 huge.

## Results

### Different TD Age Groups Show Different Signatures of Involuntary Head Motion Variability

**FIGURE 3:**
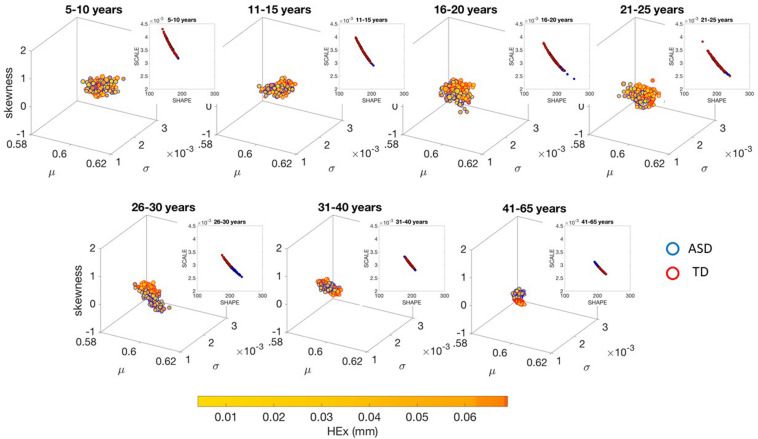
Characterization of the shifts in age-dependent stochastic signatures derived from involuntary head motion defined by linear speed (mm/s) (as in Figures 2A,B) for each of the age groups under study. Each group has equal number of representative participants (100). Gamma moment parameters (mean, standard deviation and skewness are represented by the *x*-, *y*-, and *z*-axis dimensions, respectively). The size of the marker is proportional to the kurtosis (smaller being flatter probability density function and larger being peakier distributions). The color of the marker reflects the net amount of head excursion (HEx, mm) as depicted by the colorbar gradient, while the marker’s edge color denotes the type of participant. Insets are the Gamma shape vs. scale parameter space, which we also show in Supplementary Material Figures A7, A8 in log–log units for these linear speed cases for TD and ASD, respectively.

The different age groups of TD participants showed differences in statistical signatures of NSR, with trend shifting downward with age. This result can be seen across all the age groups for the linear speed in Figure 3 and for the angular speed in the Supplementary Material Figures A1, A2 and Supplementary Table S2.

**FIGURE 4:**
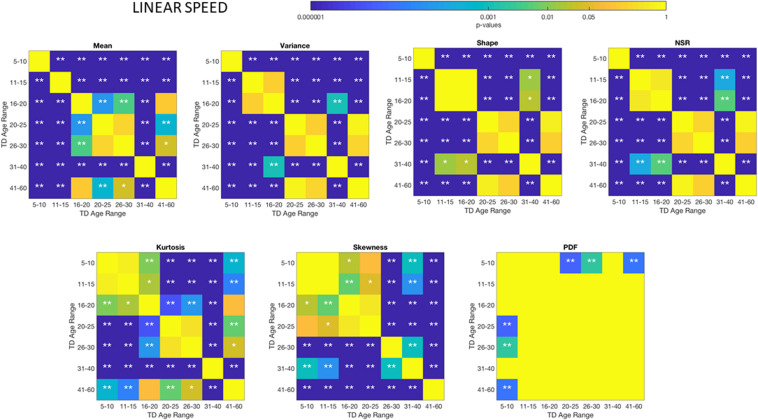
Non***−***parametric ANOVA Kruskal–Wallis pairwise statistical comparison for each TD age-group for the Gamma parameters and moments, derived from the peak amplitudes of the involuntary head motions defined by the head displacements (linear speed measured in mm/s). Reported p-values are uncorrected for multiple comparisons. *p < 0.05, **p < 0.01.

These differences in the involuntary head motions expressed by the linear speed extend to other Gamma parameters and moments in Figure 3. They reach statistical significance for all groups, as shown by Figure 4, (*p* < 0.05) when comparing pairwise each group. The NSR summarizing the variance to mean ratio is significantly different for some groups at the 0.01 level. All groups differ in NSR evolution at *p* < 0.05. In contrast the estimated PDF curves were only significantly different for 5–10 and 11–15 groups when comparing them to all the other groups; but the differences in PDF were not significant for the groups above 16 years of age. Comparable results for all parameters related to angular speed can be seen in Supplementary Material Figure A1.

### Different ASD Age Groups Show Different Signatures of Involuntary Head Motion Variability

**FIGURE 5:**
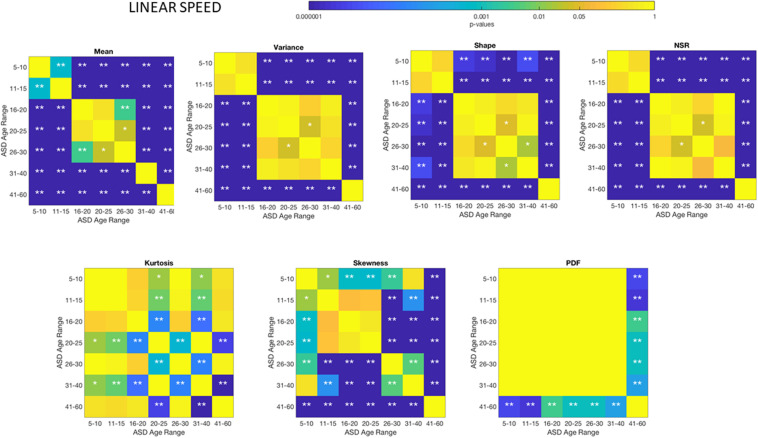
Non-parametric ANOVA ***Kruskal–Wallis*** pairwise statistical comparison for each ASD age-group for the Gamma parameters and moments, derived from the peak amplitude of involuntary head motions defined by the head displacements (linear speed measured in mm/s). Reported **p**-values are uncorrected for multiple comparisons. *p < 0.05 and **p < 0.01.

The comparisons of the age-groups with ASD also show shifting statistical signatures across ages (Figure 5) and they were significant at the 0.05 level for all comparisons in the NSR. This can be appreciated in Figure 5 for the linear speed parameter and in the Supplementary Material Figures A1–A4 for the angular speed parameter (Supplementary Table S2).

### There Are Significant Differences Between TD and ASD Groups Across Each Age-Group

**FIGURE 6:**
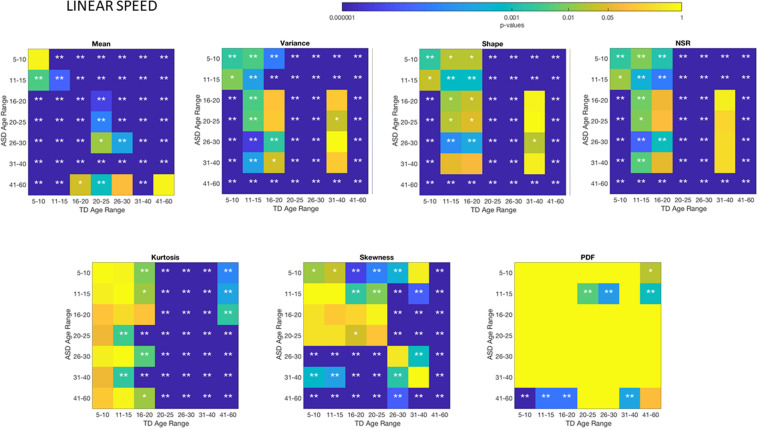
Non-parametric ANOVA ***Kruskal–Wallis*** pairwise statistical comparison for each age-group comparing TD vs. ASD for each of the Gamma parameters and moments, derived from the peak amplitude of involuntary head motions measured by the rate of displacement (linear speed mm/s). *p < 0.05 and **p < 0.01.

Differences between the age-dependent groups of TD and ASD can be appreciated in Figures 4, 5, respectively, for the linear speed. In particular, the shifts in the stochastic signatures of linear speed variability can be traced cross-sectionally across ages in the Gamma parameter space of moments, where the participants with ASD show higher variability and overall higher values of the head excursions (as quantified by the rates of linear displacements). The statistical significance of these pairwise age-group comparisons can be appreciated in the Figure 6. Further Supplementary Material Figures A1, A4 show the results corresponding to the angular speed parameter reflecting the rates of fluctuations in head rotations. Supplementary Material Figures A5, A6 further show the results for the two types of bootstrapping methods, reflecting these trends with and without replacement.

### Size Effects

The t-test for head excursions based on cumulative linear speed (head translations mm/s) yielded significant differences (*p* << 0.001) when comparing ASD and TD age groups pairwise. Likewise, the *t*-test for head excursions based on cumulative angular speed (head rotations rad/s) yielded significant differences (*p* << 0.001) when comparing ASD and TD age groups pairwise. Table 1 shows the *p*-values.

#### Disease Effect

For the comparison of ASD vs. TD, the size effects for the cumulative linear displacement of the head (head linear excursions) were in the range of very large to huge, with Glass Delta and Cohen *d*. The size effects for the cumulative angular displacements of the head (head rotational excursions) were also in the range of very large to huge, according to the Glass Delta and Cohen *d*, with the exception of age group 31–40 years old with a medium effect. Table 2 shows the effect sizes per age group.

#### Age Effects

**FIGURE 7:**
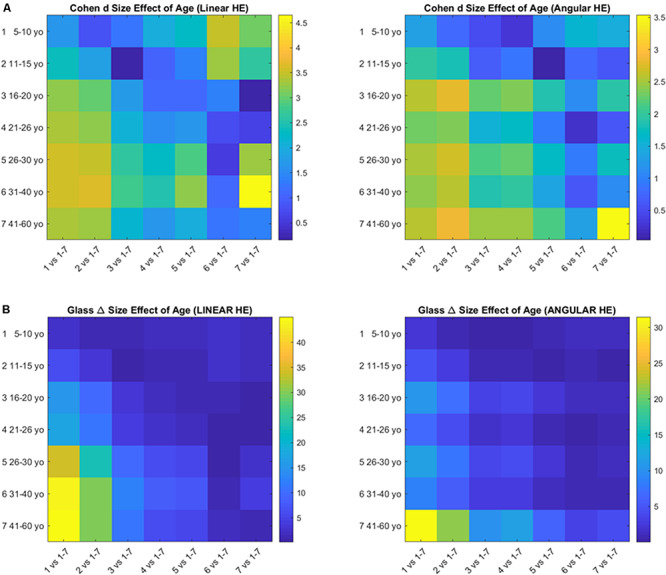
Size effects of aging according to the Cohen’s *d* **(A)** and the Glass delta **(B)** formulae applied pairwise to the age groups using the head excursion parameter derived from the cumulative linear displacements or angular rotations.

The pairwise comparison of age groups yielded large to huge size effects for the cumulative head excursions involving linear displacements or angular rotations. These effects are depicted in Figure 7 as colormaps whereby each entry of the matrix represents a pairwise age group comparison.

## Discussion

This paper investigated age-dependent shifts in the statistical signatures of typical levels of involuntary head motions using rs-fMRI data from the ABIDE repository. We characterized the stochastic signatures of involuntary head motions as TD participants rested in the scanner. We uncovered age-dependent transitions in the features of empirically estimated probability distributions of the fluctuations in peak amplitudes of linear and angular speed from involuntary head motions. We also measured the departure from this normative data in different age-groups of participants with ASD. We found that from 5 to 65 years of age, there were statistically significant differences in the distribution parameters of standardized fluctuations in speed amplitude relative to normative levels. They were paired with differences in PDF skewness and differences in PDF overall shape. We quantified mostly very large to huge size effects of these differences for disorder and age effects. The findings demonstrate that it is inadequate to assume or enforce normal distributions in statistical analyses of developmental research, including autism research. Both the linear speed and the angular speed data revealed consistent results that point at high levels of speed amplitude noise in ASD, thus making it hard to forecast future from prior speed levels.

Our work strongly suggests the need to explore age-dependent variations in noise and randomness levels in ASD motor parameters and design separate, age-appropriate analyses for young children, adolescents, and older adults. In future research, we will need to more systematically explore the typical population and build records of the age-dependent rates of change in statistical parameters reflecting levels of neuromotor control, to design new non-parametric models of normative age-shifting data. Further, our results point to the importance of studying autism as a lifelong condition that changes non-uniformly, asynchronously within a given age group and dynamically as the person ages, as compared to TD controls.

The present data set offers cross-sectional information from the ASD and TD populations. These data sets are very valuable as they revealed trends in the rates of change of probability distributions derived from *involuntary* motor data as the population ages. However, to truly characterize the heterogeneous ASD, and to stratify the population into various subtypes, we will need to deploy longitudinal studies that better reflect individual differences over time. Such differences could be tracked as the person aged and received treatments. A longitudinal and dynamic characterization of neuromotor development, including voluntary purposeful, goal-directed motions will be very important to understand the evolution of motor autonomy, action planning, action generation and action adaptation in the context of the person’s agency over naturalistic behaviors taking place in activities of daily life.

Some caveats of the ABIDE data sets are that there are different sampling resolutions of the scanners that different labs use. In recent work, we have characterized the types of noise according to sampling resolution and shown, using these same data sets, that the sampling resolution of the scanner does affect the type of noise (Caballero et al., 2018). We have also shown that the noise type can distinguish controls vs. autistic participants. Here we employed the bootstrapping technique to shuffle the speed amplitudes and randomize the possible biases that different sampling resolutions introduce. We further took care of using similar sample sizes for each age group and keeping the number of frames equal for each representative data point in the 100 set. These precautions paired with the standardization of the fluctuations deviating from an empirical estimated mean, to avoid allometric effects due to anatomical differences within a group, ensure proper comparisons. However, we also point out that breaking the groups into 5-year intervals was somewhat arbitrary, as a finer break down would have been ideal. This grouping was motivated by prior work where we were able to group medication intake and clinical scores for these groups and reveal trends across the population (Torres and Denisova, 2016). The main motivation there and here were the disparate sizes of age groups in ABIDE. We emphasize that beyond pointing out the trends in systematic shifts of probability families, we do not claim anything else. The main message of the paper is that we should not use a *one size fits all* model when performing statistical analyses, because different distributions are present in the normative groups, and in the autistic groups. Moreover, in autism, these distributions differ relative to those of controls. Levels of noise to signal ratio in these standardized waveforms systematically shift cross sectionally with aging and this reflects in a changing probability landscape that we should consider when performing our statistical analyses.

Lastly, at a different level, the results from our work are important to alert researchers, clinicians and policy makers of the shifting issues that the autistic population faces and the need for a highly flexible program that considers such shifts as the person ages. Under such profound sensory-motor differences *at the periphery* and excess of undesirable involuntary movements, it will be important to understand and characterize the types of feedback that the autistic central nervous systems are getting from the peripheral nervous systems. Once we understand these issues, we will be able to offer better support to the autistic person across all ages by leveraging sensory substitution/augmentation and noise cancelation techniques, etc. from the field of Neuroscience.

At present, autism is defined and treated as a behavioral problem reflecting issues with social interaction and communication, yet those are “*the tip of the iceberg*.” Another hidden layer of information contributing to those visible problems are these irregular micro-motions invisible to the naked eye of the diagnostician and/or the therapist. While aiming at reshaping the autistic person’s behaviors to conform to social expectations without considering such intrinsic (concealed) sensory-motor issues, the current interventions used to treat autism may unintentionally create a bigger problem.

Our lab has found that in autism, under such high levels of MMS noise across the peripheral nervous systems it is difficult to develop proper motor control (Brincker and Torres, 2013). These conclusions are supported by prior work in the field of motor control (Gidley Larson et al., 2008; Haswell et al., 2009; Marko et al., 2015; Mosconi and Sweeney, 2015; Mosconi et al., 2015a) including issues with the motor cortex (Muller et al., 2001; Theoret et al., 2005; Mostofsky et al., 2007; Floris et al., 2016; Al Sagheer et al., 2018) and the cerebellum (Mostofsky et al., 2009; Mosconi et al., 2013, 2015b). Such mounting evidence highlights the need for a better characterization of the observable behaviors defining autism in terms of underlying somatic sensory motor signatures. A neurological model (e.g., Damasio and Maurer, 1978) to explain the autistic behavioral symptoms would be more adequate to leverage the wearable sensors revolution and open a new field for *objective behavioral analyses*. Such a field would considerably help advance the neuroscience and the genetics of autism by providing new tools from AI and machine learning to automatically stratify the various subtypes of autism and guide the design of personalized treatments, accommodations and support.

One of the main features of neurotypical development is the emergence of neuromotor autonomy, which in turn depends on central control. Central control depends on the continuous peripheral feedback that kinesthetic reafferent input provides (Kandel, 2013). In neurotypical systems with intact kinesthetic feedback, mental intent matches physical action, but this is not the case in age- and sex-matched autistics (Torres et al., 2013b). This type of peripheral feedback is important for motor learning and adaptation at all levels, including socio motor behaviors, speech production via vocal apparatus and communication through pointing gestures, and gait maturation. Occupational therapists work on creating adequate support and accommodations to complete simple actions of daily living that TD individuals may take for granted, but their therapies are not always covered by medical insurance. Perhaps this type of evidence on core systemic, sensory motor differences in the autistic peripheral nervous system could help advance their programs and provide the types of objective outcome measures of treatment effectiveness that insurance companies require.

In summary, we have shown the need for new, more dynamic statistical approaches to neurodevelopment and natural aging, as well as the need to provide normative scales to measure departure from typical states in levels of motor noise, randomness and excess involuntary micro-movements in ASD.

## Data Availability Statement

The ABIDE data is publicly available. We have uploaded to ABIDE the indexes of the participants included in the current study. All methods and data types generated to produce the figures will be made available through Github, https://github.com/torreselizabeth/Frontiers-Paper.

## Ethics Statement

The studies involving data sharing from human participants were reviewed and approved by Rutgers University IRB. All data in ABIDE follows IRB approval of their corresponding university.

## Author Contributions

Conceptualization, ET methodology, ET; software, ET, CC, SM; validation, ET, CC, SM; formal analysis, ET, CC, SM; investigation, ET, CC, SM resources, ET, CC, SM data curation, CC, SM; writing—original draft preparation, ET; writing—review and editing, ET, CC, SM; visualization, ET; supervision, ET; project administration, ET; funding acquisition, ET.

## Conflict of Interest

The authors declare that the research was conducted in the absence of any commercial or financial relationships that could be construed as a potential conflict of interest.

## Footnotes

1 http://fcon_1000.projects.nitrc.org/indi/abide/abide_I.html

2 http://fcon_1000.projects.nitrc.org/indi/abide/abide_II.html

3 https://www.frontiersin.org/articles/10.3389/fnint.2018.00007/full

4 https://afni.nimh.nih.gov/pub/dist/doc/program_help/afni_proc.py.html

5 https://patents.google.com/patent/US10176299B2/en?inventor=Elizabeth+B.+TORRES

6 https://www.nature.com/articles/srep37422#Sec26
