# Supplementary material for: Age-Dependent Statistical Changes of Involuntary Head Motion Signatures Across Autism and Controls of the ABIDE Repository
Source: Front Integr Neurosci. 2020 Jun 17;14:23. doi: 10.3389/fnint.2020.00023 (PMC7311771; doi:10.3389/fnint.2020.00023)
Supplement: Supplementary file 1 [file Data_Sheet_1.pdf]

**Age-dependent Statistical Changes of Involuntary Head Motion Signatures Across Autism and Controls of the ABIDE Repository**

Carla Caballero [1,2]

Sejal Mistry [3]

Elizabeth B Torres [2,4,5*]

[1] Sports Research Center, Sports Sciences Department, Miguel Hernandez University of Elche

[2] Rutgers University, Psychology Dept., Piscataway, NJ

[3] Rutgers University, Mathematics Dept., Piscataway, NJ

[4] Computer Science, Center for Biomedical Imagining and Modeling

[5] Rutgers University Center for Cognitive Science

*Corresponding Author

ebtorres@psych.rutgers.edu

152 Frelinghuysen Rd

Piscataway, NJ 08854

# Supplementary Material Figures

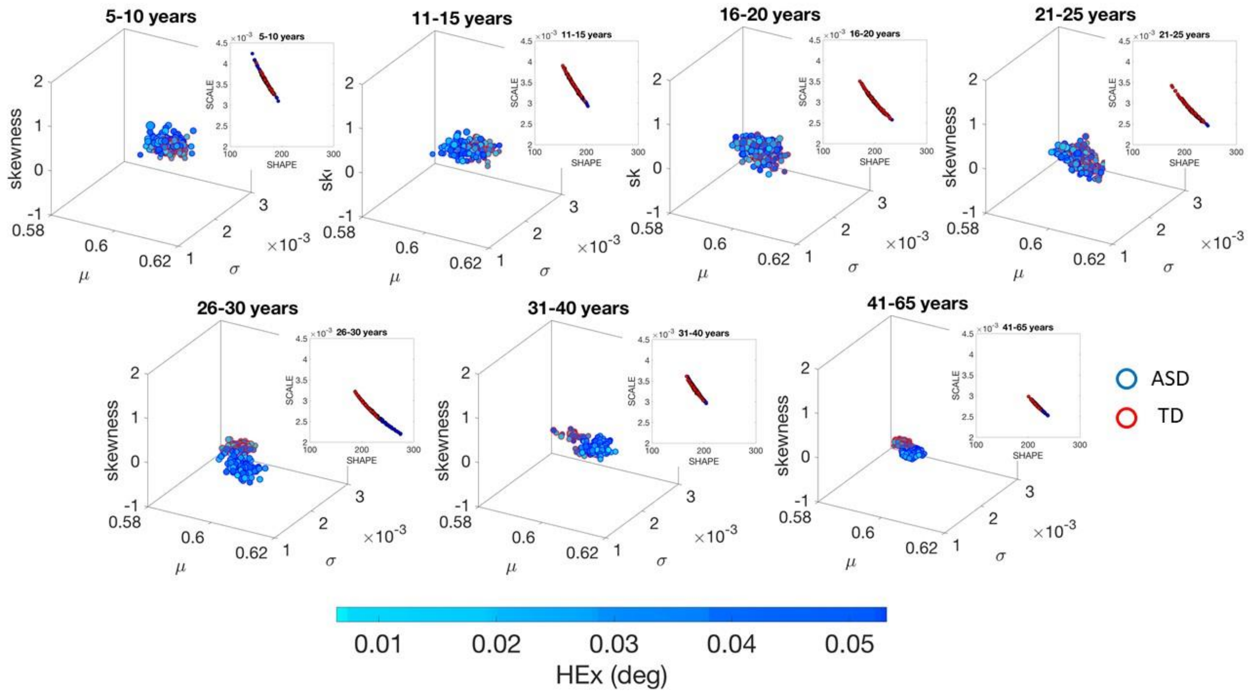


**Supplementary Material Figure 1**

Characterization of the age-dependent stochastic signatures derived from involuntary head rotations measured by angular speed (deg/s) (as in **Figure 3**) for each of the age groups under study. Each group has equal number of representative participants (100).

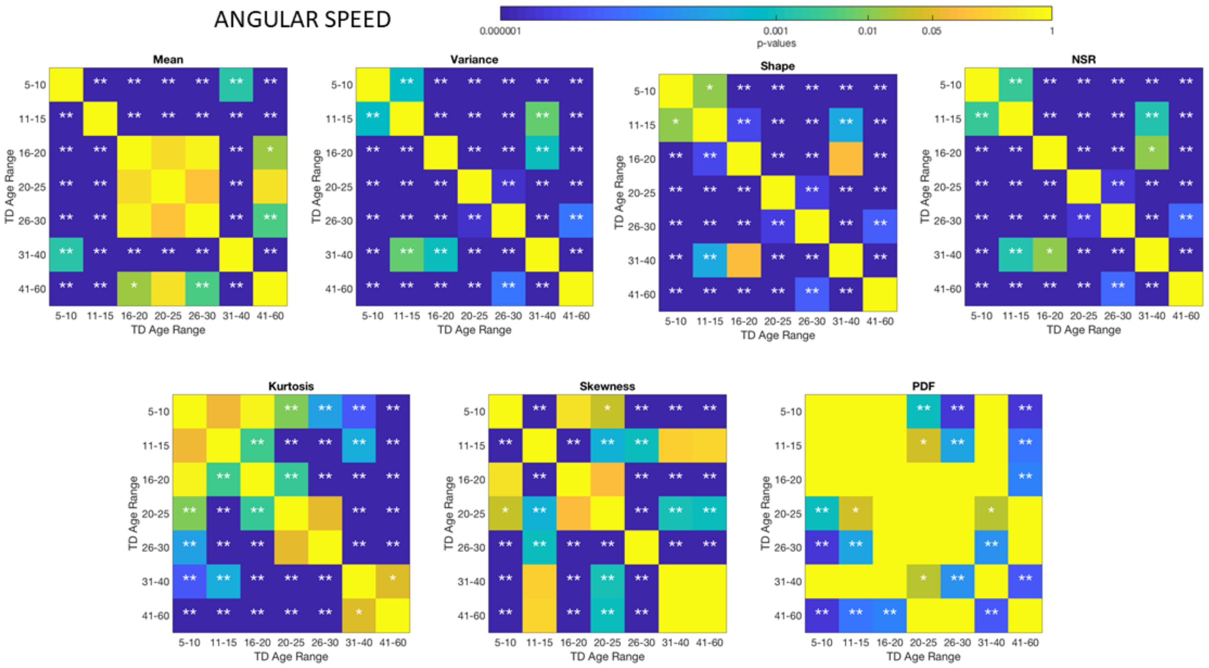


**Supplementary Material Figure 2**

Non parametric ANOVA Kruskal-Wallis pairwise statistical comparison for each TD age-group for the Gamma parameters and moments, and for the delta residual of the linear fit to the log-log Gamma parameter scatter derived from the involuntary head motions defined by the head rotations (angular speed measured in deg/s).

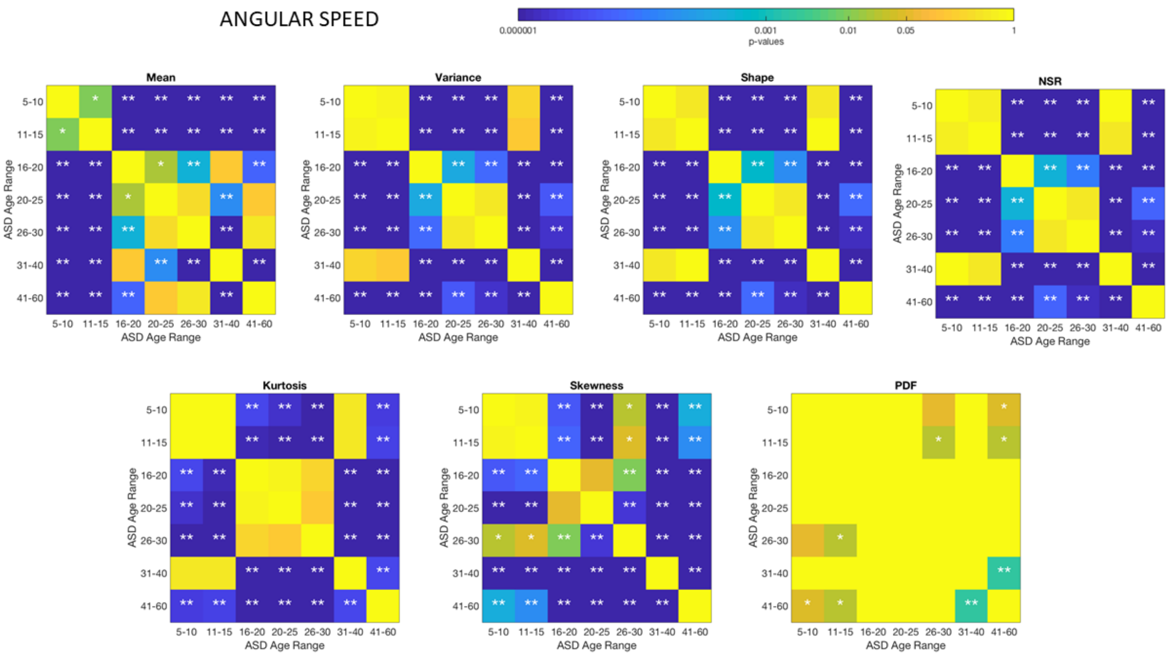


**Supplementary Material Figure 3**

Non parametric ANOVA Kruskal-Wallis pairwise statistical comparison for each age-group with ASD for the Gamma parameters and moments, and for the delta residual of the linear fit to the log-log Gamma parameter scatter derived from the involuntary head motions defined by the head rotations (angular speed measured in deg/s).

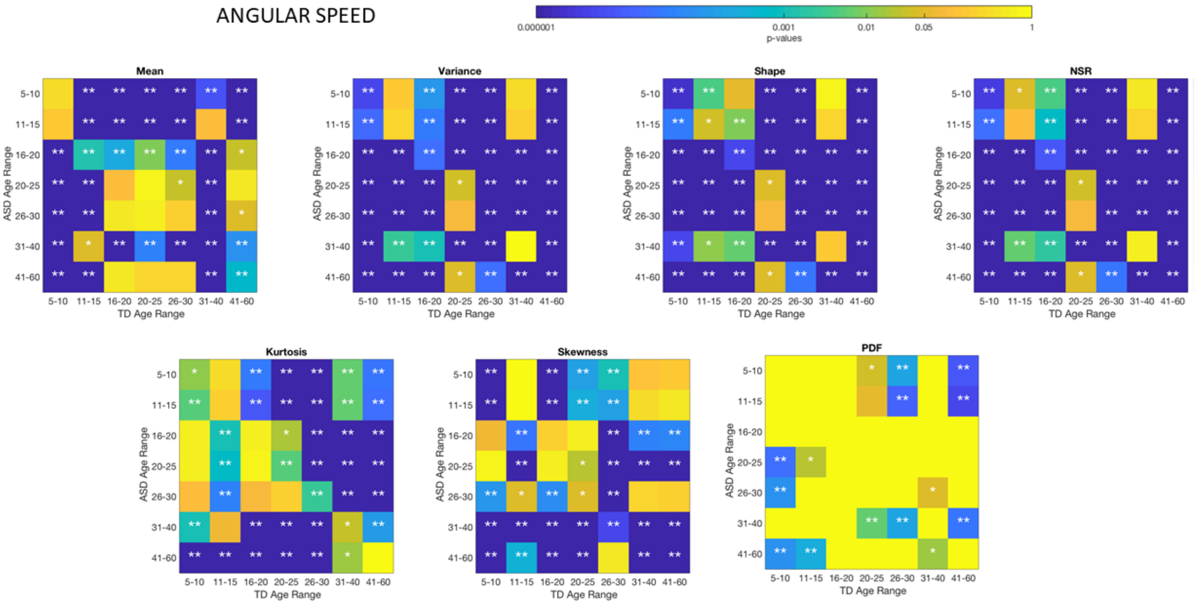


**Supplementary Material Figure 4**

Non parametric ANOVA Kruskal-Wallis pairwise statistical comparison Pairwise statistical comparison for each age-group comparing TD vs. ASD for each of the Gamma parameters and moments, and for the delta residual of the linear fit to the log-log Gamma parameter scatter derived from the involuntary head motions measured by the rate of rotation (angular speed deg/s).

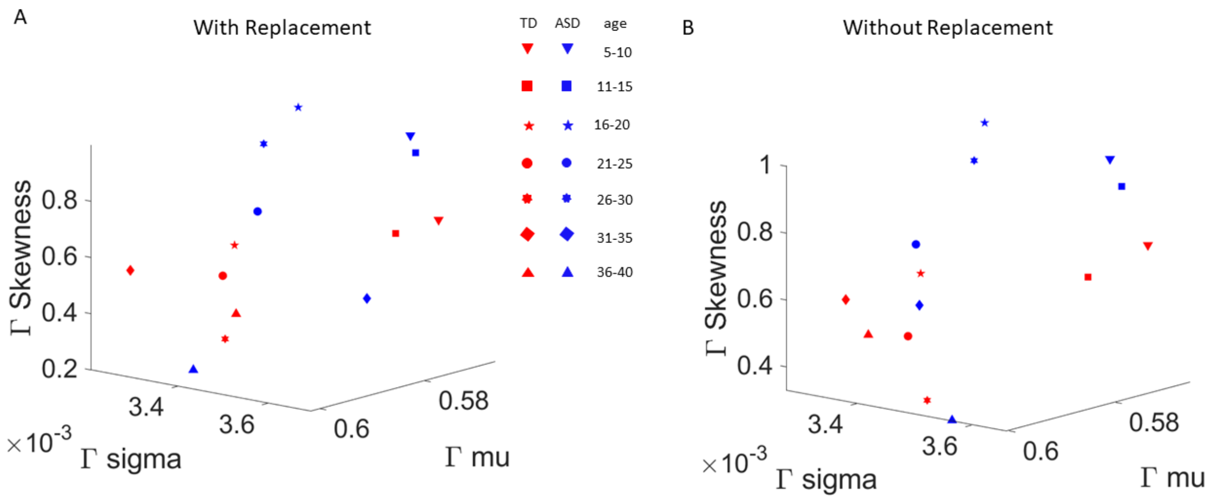


**Supplementary Material Figure 5**

Summary data obtained by pooling all participants of the age group and estimating the Gamma moments signatures for the linear displacement with and without replacement. (A) With replacement, the bootstrapping method takes 25 participants at a time from the group and cycles to 100 with replacement. (B) Without replacement, the bootstrapping method takes 25 at a time from the group and cycles through the group without replacement until it exhausts all participants in the group. Groups with fewer participants end up having fewer to bootstrap through. This affects the position of the group on the Gamma moments space but retains the relative relations between TD and ASD groups, as they remain apart in both cases. Note that no two groups overlap, indicating different distribution families per age group (as indicated by the next figure)

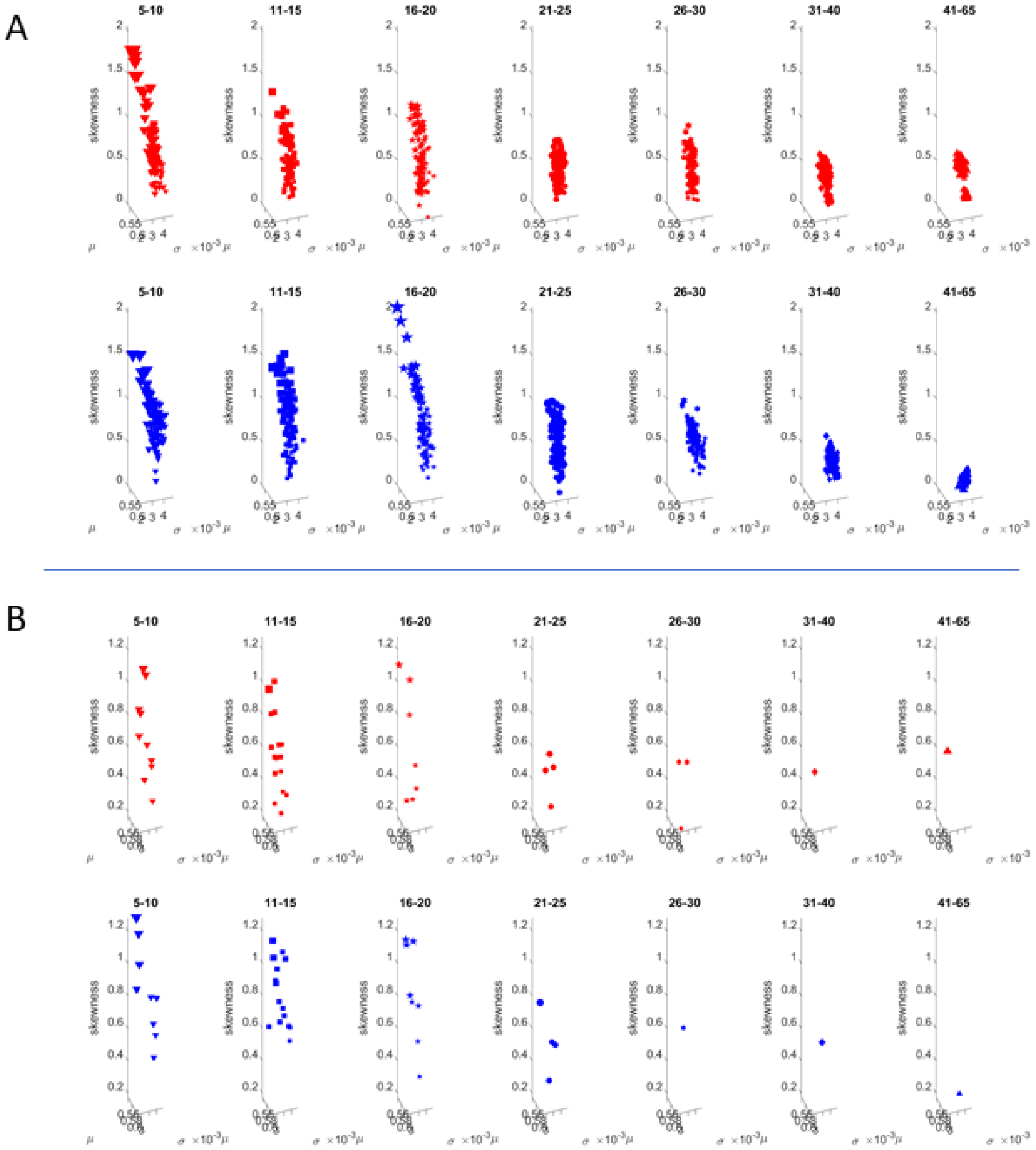


**Supplementary Material Figure 6**

Different families of Gamma distributions appear for TD age groups and for ASD age groups with different signatures that shift from age group to age group (linear displacement). (A) Sampling with replacement bootstrapping through the groups to have 100 participants in each group. (B) Sampling without replacement with fewer participants per group. The last three groups only have 1 subgroup of 25 since the sampling is without replacement and 25 cycles through the full allowed number. Table 1 shows the group sizes.

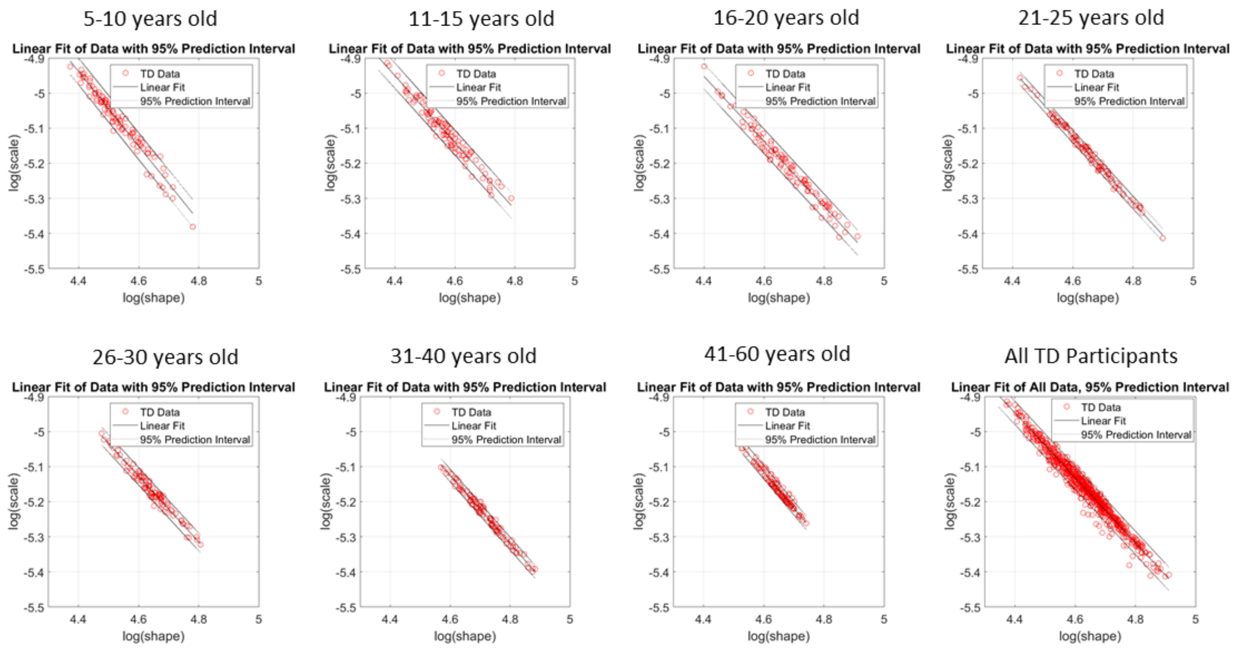


**Supplementary Material Figure 7**

| Age | Slope and Intercept | Mean Std Error Δ |
|---|---|---|
| 5-10 | -1.06 -0.24 | 0.01 |
| 11-15 | -0.95 -0.76 | 0.01 |
| 16-20 | -0.93 -0.88 | 0.01 |
| 21-25 | -0.94 -0.79 | 0.01 |
| 26-30 | -0.94 -0.82 | 0.01 |
| 31-40 | -0.97 -0.66 | 0.01 |
| 41-60 | -1.02 -0.40 | 0.01 |
| **All TD** | -0.93 -0.87 | 0.01 |

Linear polynomial fit of the log-log scatter for each of the 100 TD representatives in each age group and for the overall pooled data (last panel) with the fitted line and the 95% confidence lines. Here x is the Gamma shape value empirically estimated for each representative participant (100 points in each group and 700 in the pooled data) and y is the corresponding Gamma scale value. Delta is the standard error of the fit (i.e. the difference between the actual value and the best least square fit value y-fit), returned as a scalar value, for prediction. Generally, an interval of $y \pm \Delta$ corresponds to a roughly 68% prediction interval for future observations of large samples, and we plot here in $y \pm 2\Delta$ a roughly 95% prediction interval.

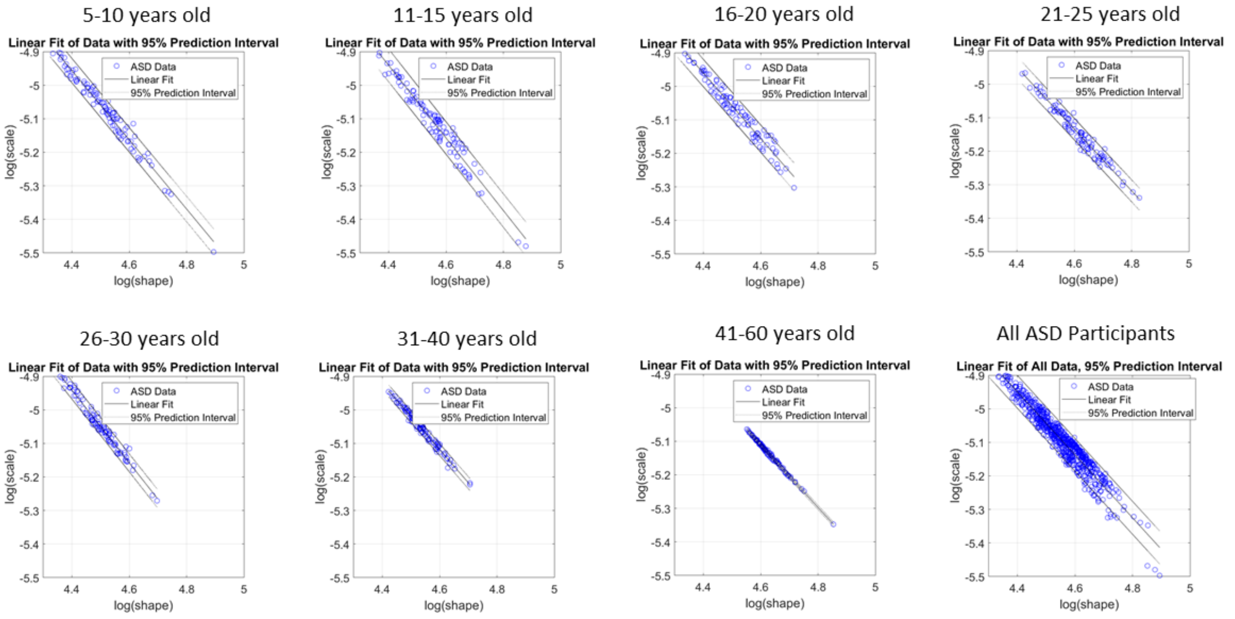


**Supplementary Material Figure 8**

| Age | Slope and Intercept | Mean Std Error Δ |
|---|---|---|
| 5-10 | -1.03 -0.41 | 0.02 |
| 11-15 | -0.98 -0.65 | 0.02 |
| 16-20 | -1.08 -0.18 | 0.02 |
| 21-25 | -0.92 -0.90 | 0.02 |
| 26-30 | -1.08 -0.17 | 0.01 |
| 31-40 | -0.99 -0.58 | 0.01 |
| 41-60 | -0.94 -0.80 | 0.02 |
| **All ASD** | -0.94 -0.81 | 0.02 |

Linear polynomial fit of the log-log scatter for each of the 100 ASD representatives in each age group and for the overall pooled data (last panel) with the fitted line and the 95% confidence lines. Data analyses as in Supplementary Material 7

***TABLE 1. NUMBER OF SUBJECTS FOR EACH GROUP EXTRACTED FROM ABIDE DATASET.***

| AGE GROUP | ASD | TD |
|---|---|---|
| **5 TO 10 YEARS OLD** | 228 | 265 |
| **11 TO 15 YEARS OLD** | 374 | 417 |
| **16 TO 20 YEARS OLD** | 200 | 178 |
| **21 TO 25 YEARS OLD** | 103 | 116 |
| **26 TO 30 YEARS OLD** | 43 | 76 |
| **31 TO 40 YEARS OLD** | 39 | 43 |
| **41 TO 65 YEARS OLD** | 30 | 32 |

***TABLE 2. ΓMEAN ± ΓSD OF THE HEAD EXCURSION FOR EACH GROUP AND EACH AGE RANGE.***

| AGE GROUP | ASD | | TD | |
|---|---|---|---|---|
| | Linear speed (mm) | Angular speed (deg) | Linear speed (mm) | Angular speed (deg) |
| **5 TO 10 YEARS OLD** | 0.0312±9.72E-05 | 0.0242±5.98E-05 | 0.0182±2.02E-05 | 0.0157±1.58E-05 |
| **11 TO 15 YEARS OLD** | 0.0231±4.32E-05 | 0.0195±2.44E-05 | 0.0135±8.63E-06 | 0.0123±5.98E-06 |
| **16 TO 20 YEARS OLD** | 0.0130±1.26E-05 | 0.0140±7.20E-06 | 0.0082±2.24E-06 | 0.0091±2.02E-06 |
| **21 TO 25 YEARS OLD** | 0.0106±67.05E-06 | 0.0147±9.19E-06 | 0.0074±1.88E-06 | 0.0103±4.31E-06 |
| **26 TO 30 YEARS OLD** | 0.0101±3.58E-06 | 0.0122±3.53E-06 | 0.0061±5.35E-07 | 0.0094±1.59E-06 |
| **31 TO 40 YEARS OLD** | 0.0065±6.63E-07 | 0.0107±2.14E-6 | 0.0057±3.29E-07 | 0.0098±2.82E-06 |
| **41 TO 60 YEARS OLD** | 0.0080±1.34E-07 | 0.0112±3.81E-07 | 0.0073±2.97E-07 | 0.0092±2.31E-07 |
